# Supplementary material for: A global scoping review of task shifting and sharing interventions to improve the mental health of people living with HIV/AIDS
Source: Glob Ment Health (Camb). 2025 May 22;12:e58. doi: 10.1017/gmh.2025.10013 (PMC12951340; doi:10.1017/gmh.2025.10013)
Supplement: Kokubun et al. supplementary material [file S2054425125100137sup001.docx]

**Supplemental File 1: Search Terms by Database**

| Search Components | PubMed/  MEDLINE (n=410) | PsycINFO (n=245) | Global Health (n=229) | Web of Science (n=448) | Scopus (n=172) |
| --- | --- | --- | --- | --- | --- |
| Population | (“people living with HIV*”[tw] OR PLWHIV[tw] OR PLWHA[tw]) | ("people living with HIV*" OR plwhiv OR plwha) | ("people living with HIV*" OR plwhiv OR plwha) | ("people living with HIV*" OR plwhiv OR plwha) (All Fields) | TITLE-ABS-KEY ("people living with HIV*" OR plwhiv OR plwha) |
| **AND** Mental health | (“Mental Health”[tw] OR "Mental Health"[Mesh] OR “Mental Illness”[tw] OR "Mental Disorders"[Majr] OR Depression[tw] OR "Depression"[Mesh] OR Anxiety[tw] OR "Anxiety"[Mesh] OR PTSD[tw] OR "Stress Disorders, Post-Traumatic"[Mesh] OR “Well-being”[tw] | ("Mental Health" OR mental AND health OR "Mental Illness" OR "Mental Disorders" OR depression OR "Depression" OR anxiety OR "Anxiety" OR ptsd OR "Stress Disorders, Post-Traumatic" OR "Well-being") | ("Mental Health" OR mental AND health OR "Mental Illness" OR "Mental Disorders" OR depression OR "Depression" OR anxiety OR "Anxiety" OR ptsd OR "Stress Disorders, Post-Traumatic" OR "Well-being") | ("Mental Health" OR mental AND health OR "Mental Illness" OR "Mental Disorders" OR depression OR "Depression" OR anxiety OR "Anxiety" OR ptsd OR "Stress Disorders, Post-Traumatic" OR "Well-being") (All Fields) | TITLE-ABS-KEY ("Mental Health" OR mental AND health OR "Mental Illness" OR "Mental Disorders" OR depression OR "Depression" OR anxiety OR "Anxiety" OR ptsd OR "Stress Disorders, Post-Traumatic" OR "Well-being") |
| **AND** Intervention | (intervention[tw] OR "Psychosocial Intervention"[Mesh] OR initiative*[tw] OR program*[tw] OR programme*[tw] OR “community-based participatory research”[tw] OR "Community-Based Participatory Research/methods"[Mesh]) | (intervention OR "Psychosocial Intervention" OR initiative* OR program* OR programme* OR "community-based participatory research" OR "Community-Based Participatory Research/methods") | (intervention OR "Psychosocial Intervention" OR initiative* OR program* OR programme* OR "community-based participatory research" OR "Community-Based Participatory Research/methods") | (intervention OR "Psychosocial Intervention" OR initiative* OR program* OR programme* OR "community-based participatory research" OR "Community-Based Participatory Research/methods") (All Fields) | TITLE-ABS-KEY (intervention OR "Psychosocial Intervention" OR initiative* OR program* OR programme* OR "community-based participatory research" OR "Community-Based Participatory Research/methods") |
| **AND** Task shifting and task sharing | (“task shift*”[tw] OR “task shar*”[tw] OR “task-shift*”[tw] OR “task-shar*”[tw] OR “lay-person*”[tw] OR layperson*[tw] OR “lay people”[tw] OR “laypeople”[tw] OR “lay-people”[tw] OR “Lay Health”[tw] OR voluntary[tw] OR volunteer*[tw] OR non-professional*[tw] OR nonprofessional*[tw] OR non-specialist*[tw] OR paraprofessional*[tw] OR peer*[tw] OR "Peer Group"[Mesh] OR “Community Health Worker*”[tw] OR “community healthcare worker*”[tw] OR “social support*”[tw] OR "Social Support"[Mesh] OR “Psychosocial Support*”[tw] OR "Psychosocial Support Systems"[Mesh] OR MHPSS[tw] OR cadre*[tw]) | ("task shift*" OR "task shar*" OR "task-shift*" OR "task-shar*" OR "lay-person*" OR layperson* OR "lay people" OR "laypeople" OR "lay-people" OR "Lay Health" OR voluntary OR volunteer* OR non-professional* OR nonprofessional* OR non-specialist* OR paraprofessional* OR peer* OR "Peer Group" OR "Community Health Worker*" OR "community healthcare worker*" OR "social support*" OR "Social Support" OR "Psychosocial Support*" OR "Psychosocial Support Systems" OR mhpss OR cadre*) | ("task shift*" OR "task shar*" OR "task-shift*" OR "task-shar*" OR "lay-person*" OR layperson* OR "lay people" OR "laypeople" OR "lay-people" OR "Lay Health" OR voluntary OR volunteer* OR non-professional* OR nonprofessional* OR non-specialist* OR paraprofessional* OR peer* OR "Peer Group" OR "Community Health Worker*" OR "community healthcare worker*" OR "social support*" OR "Social Support" OR "Psychosocial Support*" OR "Psychosocial Support Systems" OR mhpss OR cadre*) | ("task shift*" OR "task shar*" OR "task-shift*" OR "task-shar*" OR "lay-person*" OR layperson* OR "lay people" OR "laypeople" OR "lay-people" OR "Lay Health" OR voluntary OR volunteer* OR non-professional* OR nonprofessional* OR non-specialist* OR paraprofessional* OR peer* OR "Peer Group" OR "Community Health Worker*" OR "community healthcare worker*" OR "social support*" OR "Social Support" OR "Psychosocial Support*" OR "Psychosocial Support Systems" OR mhpss OR cadre*) (All Fields) | TITLE-ABS-KEY ("task shift*" OR "task shar*" OR "task-shift*" OR "task-shar*" OR "lay-person*" OR layperson* OR "lay people" OR "laypeople" OR "lay-people" OR "Lay Health" OR voluntary OR volunteer* OR non-professional* OR nonprofessional*  OR non-specialist* OR paraprofessional* OR peer* OR "Peer Group" OR "Community Health Worker*" OR "community healthcare worker*" OR "social support*" OR "Social Support" OR "Psychosocial Support*" OR "Psychosocial Support Systems" OR mhpss OR cadre*) |

Note: The n’s reported for each database reflect the number of search returns at the time the search was conducted.
